# Gilles de la Tourette syndrome as a rare co-morbidity of Klinefelter syndrome

**DOI:** 10.1007/s10072-024-07573-x

**Published:** 2024-05-07

**Authors:** Andrea E. Cavanna, Giulia Paini, Giulia Purpura, Anna Riva, Renata Nacinovich, Stefano Seri

**Affiliations:** 1 Department of Neuropsychiatry, BSMHFT and University of Birmingham, Birmingham, UK; 2 Sobell Department of Motor Neuroscience and Movement Disorders, Institute of Neurology, University College London, London, UK; 3 School of Health and Life Sciences, Aston Institute of Health and Neurodevelopment, Aston University, Birmingham, UK; 4 School of Medicine and Surgery, University of Milano-Bicocca, Milan, Italy; 5 Department of Child Neuropsychiatry, IRCCS San Gerardo dei Tintori, Monza, Italy; 6 Department of Neuropsychiatry, National Centre for Mental Health, 25 Vincent Drive, Birmingham, B15 2FG UK

**Keywords:** Klinefelter syndrome, XXY, Tics, Tourette syndrome

## Abstract

**Background:**

Klinefelter syndrome (47, XXY) is the most common sex chromosome aneuploidy. In addition to male hypergonadotropic hypogonadism, a wide range of neurodevelopmental disorders, anxiety and affective symptoms have been reported in a substantial proportion of cases.

**Case description:**

We document the rare case of a 43-year-old man diagnosed with Klinefelter syndrome and co-morbid Gilles de la Tourette syndrome. He presented with multiple motor and vocal tics since adolescence, as well as anxiety and affective symptoms as his main tic-exacerbating factors. Tic severity was rated as marked (Yale Global Tic Severity Scale score of 78/100), and recommendations for the treatment of both tics and psychiatric co-morbidities were formulated.

**Discussion:**

Neurodevelopmental tics in the context of Klinefelter syndrome have been previously documented in three cases only. Gilles de la Tourette syndrome is 3–4 times more common in males than females and its etiological factors include multiple genetic components (genetic heterogeneity). Our case report widens the spectrum of neurodevelopmental disorders observed in the context of Klinefelter syndrome and contributes to genetic research on the role of the X chromosome in the pathophysiology of tic disorders.

## Introduction

Klinefelter syndrome (KS) is the most common sex chromosome aneuploidy (47, XXY) and cause of male hypergonadotropic hypogonadism. It is estimated that 0.1–0.2% of newborn males have an extra X chromosome, resulting in a syndrome characterized by high clinical heterogeneity [1]. In addition to hypogonadism, infertility, language delay and metabolic co-morbidities, subjects with KS can present with a range of neuropsychiatric disorders. Specifically, neurodevelopmental disorders (autism spectrum disorder, attention-deficit and hyperactivity disorder), anxiety and affective symptoms have been reported in a substantial proportion of cases [2].

Gilles de la Tourette syndrome (GTS) is a neurodevelopmental disorder characterized by the presence of multiple motor and vocal tics, with onset before the age of 18 [3]. GTS affects 0.4-1% of the population, and most subjects with GTS present with co-occurring conditions, especially tic-related obsessive-compulsive symptoms and other neurodevelopmental conditions [4]. Here, we provide the rare report of a patient with KS associated with GTS.

## Case report

A.B., a 43-year-old man with KS, was referred to the specialist Tourette syndrome Clinic, Department of Neuropsychiatry, BSMHFT and University of Birmingham (United Kingdom), for the assessment and further management of multiple motor and vocal tics. A.B. was diagnosed with KS associated with male hypogonadism and non-insulin dependent diabetes. He also reported a longstanding history of tic symptoms, dating back to adolescence. The age at tic onset was 15, and the first tic to be noticed was a neck jerk. He subsequently developed multiple motor tics, including eye blinking, eye squeezing, mouth opening, mouth pulling, jaw clenching, facial grimacing, spitting, head nodding (backwards), hair-out-of-the-eyes flicking movements, neck stretching, arm extending, arm crossing, finger stretching, gluteus tensing, kicking, foot tapping, stamping, and motor tics resulting in abnormal gait (taking longer steps). In terms of vocal tics, he reported grunting, coughing, barking, snorting, shouting, squeaking, vowel (‘ah’) sounds, and talking to himself. As for complex tics and tic-related symptoms, A.B. reported coprolalia, echopraxia, forced touching, and other socially inappropriate behaviours, including random words. In addition, he reported tic-related self-injurious behaviours, including self-hitting at the level of his head and chest.

A.B.’s tics were described as being characteristically preceded by premonitory urges and temporarily suppressible at the expense of mounting inner tension. Psychological stress was singled out as the main tic-exacerbating factor, whereas physical activity and active concentration were listed as tic-alleviating factors. Over time, A.B.’s tics followed a waxing and waning course, with a relapse over the months preceding his referral to the specialist Tourette syndrome Clinic. A.B. did not report the presence of overt tic-related obsessive-compulsive symptoms, apart from mild ‘just right’ perceptions and obsessional thoughts in the form of mental images with distressing content. A.B. described multimodal (tactile, auditory) hypersensitivity, but he did not receive a formal diagnosis of autism spectrum disorder and there was no clear history of childhood attention-deficit and hyperactivity disorder. There was a longstanding history of anxiety and affective symptoms (including mood swings), as well as sleep problems.

On neurological examination, there was evidence of both motor and vocal tics; mental state examination revealed elevated levels of anxiety. Both birth and early development were reported as normal. A.B. did not develop stuttering or learning disabilities and received mainstream education. A.B.’s pharmacotherapy included Haloperidol 3 mg daily, Citalopram 40 mg daily, Metformin 1500 mg daily, and testosterone injections every 16 weeks for hypogonadism. He reported previous trials on Sulpiride and Aripiprazole, which were discontinued because of lack of efficacy against tics and poor tolerability, respectively.

There was a family history of mild motor tics (father) and obsessive-compulsive symptoms (paternal uncle). A.B. scored 86% on the Tourette syndrome Diagnostic Confidence Index (which is above the average scores reported at specialist clinics) and 78% on the Yale Global Tic Severity Scale, indicating marked tic severity. In relation to behavioural co-morbidities, there was evidence of underlying anxiety and affective symptoms as main tic-exacerbating factors. In addition to confirming the diagnosis of GTS, specialist advice was provided, with recommendations for treatment interventions. These included further pharmacotherapy options (add-on Topiramate up to 200 mg daily, Clonidine up to 0.2 mg daily), as well as cognitive-behavioural therapy for underlying anxiety and affective symptoms.

## Discussion

To the best of our knowledge, there are only two previous reports of co-occurring GTS and KS, despite the increased prevalence of neurodevelopmental co-morbidities in both conditions [2, 4]. The first report dates back to 1969, when two boys with prepubertal KS and tics alongside anxiety and other psychiatric symptoms were admitted to the Hesperia Hospital in Helsinki [5]. More recently, a 20-year old male with KS was referred for evaluation of tics to the specialist Center for Movement Disorders in Gainesville, Florida [6]. His motor tics began at the age of 4 with repetitive eye fluttering, progressing over the years to a constellation of motor and vocal tics including painful head jerking, limb flailing, kicking, humming, nose clearing, shouting, and uttering words. Of note, his school performance was affected by co-morbid attention-deficit and hyperactivity disorder.

Tic disorders, including GTS, are 3–4 times more common in males than females, and are characterized by genetic heterogeneity [3]. Rare X-linked variants carry predominantly male risk in neurodevelopmental conditions, including GTS [7]. In a previous report, a microduplication at Xq21.31 was present in three brothers with GTS including the proband, but not in an unaffected brother [8]. Interestingly, the AKAP17A gene on the X chromosome codes for a kinase A anchoring protein that is upregulated in individuals with KS and has been associated with chronic tic disorders [9]. It has been suggested that cases of KS are associated with disturbances in basic biological pathways related to cell growth and in mRNA splicing as well as an increased susceptibility to develop tic disorders [10].

In conclusion, we have presented a rare case of KS associated with GTS, widening the spectrum of neurodevelopmental disorders observed in the context of KS and contributing to research avenues focusing on the role of the X chromosome in the pathophysiology of tic disorders.
